# Adamantine Amantadine

**DOI:** 10.1007/s11606-025-09412-x

**Published:** 2025-04-24

**Authors:** Lealani Mae Y. Acosta

**Affiliations:** Department of Neurology, Vanderbilt University Medical Center, Nashville, TN USA

## Abstract

Recognition of the serendipitous history of amantadine, first as a flu treatment and now as a Parkinson disease drug, and its adamantine persistence, and how small changes, like rearranging a few letters in a word or a DNA strand, can have a big impact.

adamantine (adj.)^1^

1. : made of or having the quality of *adamant*
2. : rigidly firm: UNYIELDING
3. : resembling the diamond in hardness or luster

amantadine (n.)

1. :blocks viral particle uncoating and nucleic acid release into host cell, inhibiting viral replication
2. :exact mechanism in Parkinson’s disease unknown
3. :potentiates CNS dopaminergic response

The virus graphic in my middle school life science class

had a *diamanté* shaped head, teetering on spidery legs.

Influenza is not diamond-shaped, though:

flu, whether spherical or filamentous,

has spikes that penetrate,^2^ ultimately yielding to amantadine.

Yet amantadine itself yielded to progress:

its—ta-da!—disappeared in the hocus pocus of mutant bugs,

now superseded in flu prophylaxis and treatment by

new drugs that loosened flu’s grip

then the hand of fate shook with serendipity

rigidity loosened, tremor abated in a patient with Parkinson’s and the flu^3^

from this eve of a new era, adamant researchers recruited more Adams and Eves,

man and woman,

clinical research cohorts of men and women

cogs, wheels turned

amygdalas mediating excitement at this new application

carrying the mantle alone or with other drugs:

a few letters substituted

like codons in a DNA strand, like the swipe of a scythe from glutamic acid to valine

missense, nonsense,

frameshift of perspective

no use to new use

adamantine amantadine
